# Unmasking a Hemangioblastoma: A Case of Obstructive Hydrocephalus in a 43-Year-Old Male Patient

**DOI:** 10.7759/cureus.82746

**Published:** 2025-04-21

**Authors:** Ali M Rida, Caleb M Glover, Eric Nguyen, Scott Plaehn

**Affiliations:** 1 Internal Medicine, Michigan State University, Lansing, USA; 2 Internal Medicine, McLaren Greater Lansing, Lansing, USA; 3 Gastroenterology, McLaren Greater Lansing, Lansing, USA

**Keywords:** abdominal pain, brain tumor, hemangioblastoma, nausea and vomiting, obstructive hydrocephalus

## Abstract

This case report discusses a 43-year-old male patient with a history of chronic kidney disease and gastroesophageal reflux disease (GERD) who presented with nonspecific symptoms, including nausea, vomiting, abdominal discomfort, and mild headaches. Initially attributed to gastrointestinal issues, further investigation revealed a 1 cm enhancing mass in the fourth ventricle via imaging, causing obstructive hydrocephalus. A successful suboccipital craniotomy was performed to resect the hemangioblastoma, a highly vascular tumor. The patient had an uneventful recovery and was scheduled for follow-up to monitor for potential recurrence and associated conditions, such as those related to von Hippel-Lindau syndrome. This case highlights the critical importance of considering brain tumors in patients with vague presenting symptoms and reinforces the role of timely imaging and surgical intervention in improving patient outcomes.

## Introduction

Brain tumors, though relatively rare, remain a significant cause of morbidity and mortality worldwide. Their symptoms can be nonspecific and often mimic other common conditions, making early diagnosis challenging. Common manifestations include headaches, seizures, focal neurological deficits, altered mental status, and nausea or vomiting [1,2]. These symptoms are frequently attributed to a variety of gastrointestinal, vascular, or metabolic conditions, which can delay the identification of a neurological issue [3].

Imaging studies, particularly computed tomography (CT) and magnetic resonance imaging (MRI), are pivotal in diagnosing these tumors, providing detailed views of intracranial structures. Among the various types of brain tumors, vascular tumors like hemangioblastomas are rare but significant due to their potential to cause obstructive hydrocephalus and neurological deficits [4,2]. Hemangioblastomas are typically highly vascular and benign, with the ability to significantly impact brain function if left untreated. They are often found in the cerebellum and fourth ventricle and are associated with conditions such as von Hippel-Lindau disease (VHL) [3]. Surgical intervention is often required, with resection being the primary treatment option. Understanding these tumors' clinical presentation, diagnostic process, and management is crucial for timely intervention and optimal patient outcomes [4,2].

## Case presentation

The patient, a 43-year-old male with a history of chronic kidney disease (Not on Dialysis, baseline Cr. of 1.4), chronic history of anisocoria Left > Right from left orbital trauma seven years prior, and chronic gastroesophageal reflux disease (GERD; on famotidine 20mg twice daily at home), who initially presented with complaints of nausea, vomiting, epigastric discomfort, poor appetite, and intermittent mild headaches, which he had been experiencing for the past two weeks. These symptoms were initially attributed to food poisoning during overseas travel. Upon his return, the nausea, vomiting, and epigastric pain persisted, accompanied by poor concentration, fatigue, and difficulty with ambulation.

Initial workup included a CT scan of the abdomen and pelvis, which revealed no acute abdominal process. An abdominal ultrasound was performed and was negative for gallstones or any other abnormal findings. Endoscopy showed normal mucosal appearance without evidence of gastritis, ulcers, or *Helicobacter pylori *infection. A coronary CT angiography, performed previously, was unremarkable. Despite symptomatic treatment, including IV fluids, Zofran (ondansetron) for nausea, Pepcid (famotidine) for gastric protection, and Toradol (ketorolac) for pain relief, the patient’s symptoms persisted. Further treatment included Carafate (sucralfate) for ongoing discomfort.

**Figure 1:**
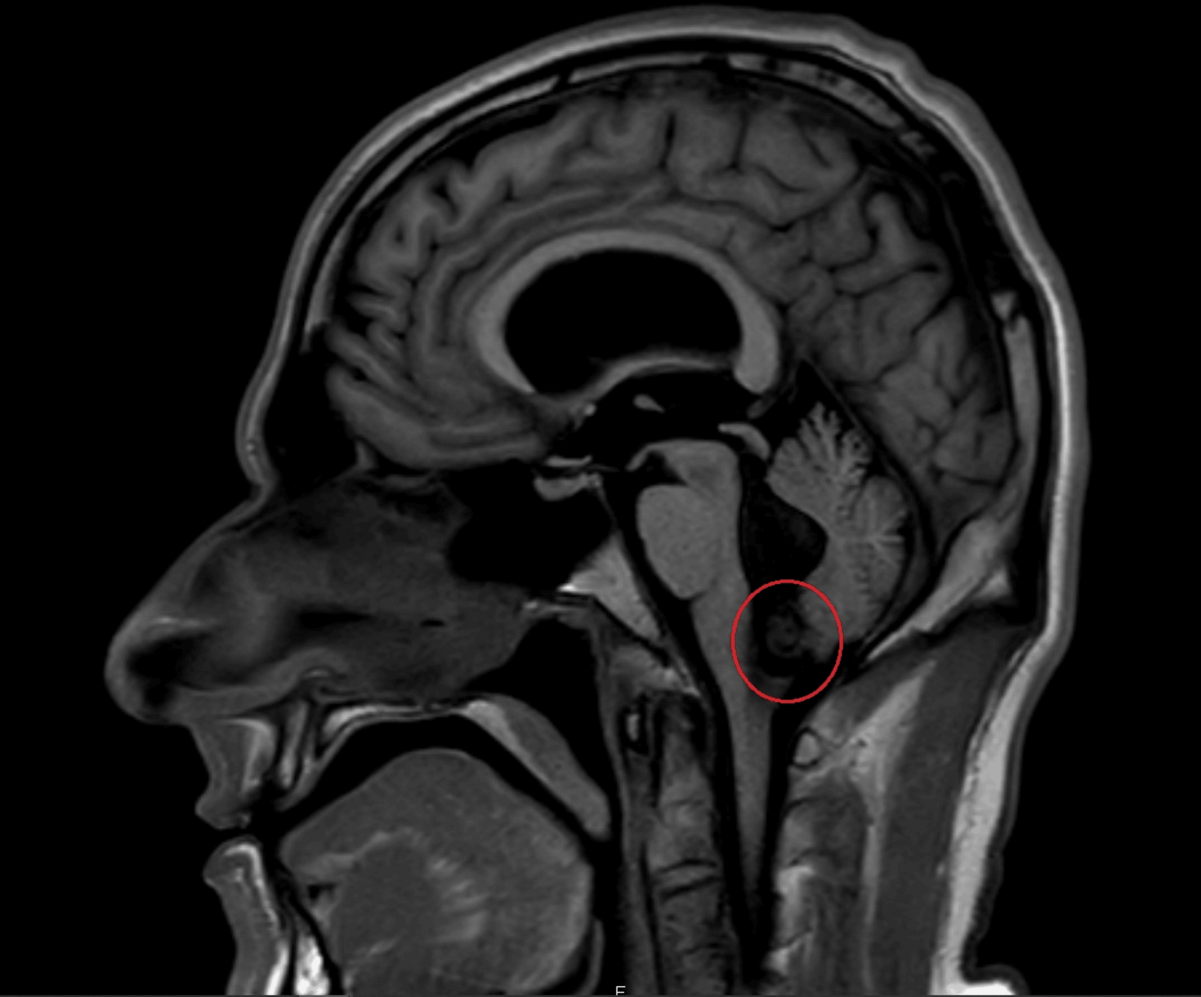
MRI head demonstrating a 1 cm intraventricular mass within the fourth ventricle at the foramen of Magendie resulting in moderate obstructive hydrocephalus (red circle).

The patient was transferred to a second facility for further evaluation, where he continued to experience nausea, vomiting, epigastric pain, and mild headaches. A CT scan of the head revealed a 1 cm enhancing mass located in the fourth ventricle at the foramen of Magendie, causing obstructive hydrocephalus and moderate ventriculomegaly. These findings were suggestive of a space-occupying lesion obstructing cerebrospinal fluid (CSF) flow. The MRI confirmed these findings, with a homogeneous, enhancing lesion in the fourth ventricle, measuring approximately 13.1 x 12.6 x 14.1 mm, resulting in moderate to severe obstructive hydrocephalus (Figure 1). There was no evidence of tonsillar herniation or acute infarction, and the ventriculomegaly appeared stable, with minimal transependymal CSF flow, indicating chronicity rather than acute hydrocephalus. A CT scan with contrast of the cervical spine was performed and was negative for any acute abnormalities.

Given the findings of the fourth ventricular mass and obstructive hydrocephalus, a neurosurgical consultation was requested. The mass was highly suspicious for a choroid plexus papilloma, though ependymoma was also considered. The patient was transferred to the Neurosciences Intensive Care Unit for further management and preoperative preparation.

On the day of surgery, the patient underwent a suboccipital craniotomy for resection of the fourth ventricular mass. Intraoperative findings confirmed the mass was highly vascular, with multiple blood vessels supplying it, consistent with a hemangioblastoma. The mass was successfully removed in one piece without damaging the underlying brainstem. Neurophysiological monitoring during surgery was stable, and the procedure was well tolerated.

A follow-up MRI was performed to assess the status of the brain tumor resection, and the patient’s recovery was progressing without any complications. Post-surgery, the patient had significant improvement in his symptoms.

Upon discharge, the patient was instructed to follow up with neurosurgery for continued monitoring. Follow-up appointments were scheduled with the primary neurosurgeon and with ophthalmology for possible retinal involvement, given his chronic history of anisocoria. He was also referred for audiology consultation and additional imaging to rule out other neuroendocrine sources, including a possible renal cell carcinoma (RCC), pheochromocytoma, paraganglioma, or pancreatic neuroendocrine tumors.

## Discussion

The diagnostic process for brain tumors begins with a comprehensive clinical evaluation. In many cases, nonspecific symptoms such as headache, nausea, vomiting, and fatigue can be mistaken for gastrointestinal or systemic disorders. Imaging modalities like CT and MRI are essential for detecting space-occupying lesions and providing detailed information about the tumor’s size, location, and characteristics. In the case of obstructive hydrocephalus, the imaging results are crucial for identifying the tumor’s effect on CSF flow, which is often the primary cause of the condition. The diagnosis of a hemangioblastoma, in particular, requires consideration of the tumor’s vascular nature, which is often apparent in both imaging and intraoperative findings. These tumors are known for their rich blood supply, which can complicate surgical resection and necessitate careful planning during surgery [5].

Hemangioblastomas are rare benign tumors most commonly found in the cerebellum and fourth ventricle, but they can also be located in other areas of the central nervous system. Although benign, they can cause significant neurological impairment due to their tendency to compress surrounding structures and obstruct CSF flow. Surgical resection remains the treatment of choice, as it offers the best chance for symptom relief and long-term survival. In this case, the patient underwent a suboccipital craniotomy with successful resection of the tumor. Postoperative recovery was uneventful, highlighting the effectiveness of surgical management. However, long-term follow-up is necessary to monitor for recurrence and associated conditions, including potential systemic manifestations like retinal hemangiomas, which are often seen in patients with VHL syndrome [5,6].

## Conclusions

This case highlights the importance of considering brain tumors in the differential diagnosis of patients presenting with nonspecific symptoms such as headache, nausea, and vomiting. Imaging studies are critical in identifying space-occupying lesions and guiding appropriate management. Hemangioblastomas, though rare, should be considered in cases involving vascular tumors of the central nervous system, especially when obstructive hydrocephalus is present. Surgical resection offers excellent outcomes for most patients, although ongoing surveillance is crucial to detect any recurrence or associated conditions. Timely diagnosis and treatment are key to optimizing patient prognosis and quality of life.
